# Autonomic response to walk tests is useful for assessing outcome measures in people with multiple sclerosis

**DOI:** 10.3389/fphys.2023.1145818

**Published:** 2023-04-06

**Authors:** Spyridon Kontaxis, Estela Laporta, Esther Garcia, Ana Isabel Guerrero, Ana Zabalza, Martinis Matteo, Roselli Lucia, Sara Simblett, Janice Weyer, Matthew Hotopf, Vaibhav A. Narayan, Zulqarnain Rashid, Amos A. Folarin, Richard J. B. Dobson, Mathias Due Buron, Letizia Leocani, Nicholas Cummins, Srinivasan Vairavan, Gloria Dalla Costa, Melinda Magyari, Per Soelberg Sørensen, Carlos Nos, Raquel Bailón, Giancarlo Comi, the RADAR-CNS Consortium

**Affiliations:** ^1^ Laboratory of Biomedical Signal Interpretation and Computational Simulation (BSICoS), University of Zaragoza, Zaragoza, Spain; ^2^ Biomedical Research Networking Center in Bioengineering, Biomaterials and Nanomedicine (CIBER-BBN), Barcelona, Spain; ^3^ Department of Microelectronics and Electronic Systems, Autonomous University of Barcelona, Barcelona, Spain; ^4^ Multiple Sclerosis Centre of Catalonia (CEMCAT), Department of Neurology/Neuroimmunology, Hospital Universitari Vall D’Hebron, Universitat Autónoma de Barcelona, Barcelona, Spain; ^5^ Faculty of Medicine and Surgery, Vita Salute San Raffaele University, Milan, Italy; ^6^ Institute of Psychiatry, Psychology and Neuroscience, King’s College London, London, United Kingdom; ^7^ RADAR-CNS Patient Advisory Board, London, United Kingdom; ^8^ Research and Development Information Technology, London, United Kingdom; ^9^ Department of Biostatistics and Health Informatics, Institute of Psychiatry, Psychology and Neuroscience, King’s College London, London, United Kingdom; ^10^ Institute of Health Informatics, University College London, London, United Kingdom; ^11^ Danish Multiple Sclerosis Centre, Department of Neurology, Copenhagen University Hospital Rigshospitalet, Copenhagen, Denmark; ^12^ Casa di Cura del Policlinico, Milan, Italy; ^13^ The RADAR-CNS Consortium, London, United Kingdom

**Keywords:** autonomic nervous system, heart rate variability, ECG-derived respiration, relapsing-remitting multiple sclerosis, secondary progressive multiple sclerosis, fatigue, disability, walking capacity

## Abstract

**Objective:** The aim of this study was to evaluate the association between changes in the autonomic control of cardiorespiratory system induced by walk tests and outcome measures in people with Multiple Sclerosis (pwMS).

**Methods:** Electrocardiogram (ECG) recordings of 148 people with Relapsing-Remitting MS (RRMS) and 58 with Secondary Progressive MS (SPMS) were acquired using a wearable device before, during, and after walk test performance from a total of 386 periodical clinical visits. A subset of 90 participants repeated a walk test at home. Various MS-related symptoms, including fatigue, disability, and walking capacity were evaluated at each clinical visit, while heart rate variability (HRV) and ECG-derived respiration (EDR) were analyzed to assess autonomic nervous system (ANS) function. Statistical tests were conducted to assess differences in ANS control between pwMS grouped based on the phenotype or the severity of MS-related symptoms. Furthermore, correlation coefficients (*r*) were calculated to assess the association between the most significant ANS parameters and MS-outcome measures.

**Results:** People with SPMS, compared to RRMS, reached higher mean heart rate (HRM) values during walk test, and larger sympathovagal balance after test performance. Furthermore, pwMS who were able to adjust their HRM and ventilatory values, such as respiratory rate and standard deviation of the ECG-derived respiration, were associated with better clinical outcomes. Correlation analyses showed weak associations between ANS parameters and clinical outcomes when the Multiple Sclerosis phenotype is not taken into account. Blunted autonomic response, in particular HRM reactivity, was related with worse walking capacity, yielding *r* = 0.36 *r* = 0.29 (RRMS) and *r* > 0.5 (SPMS). A positive strong correlation *r* > 0.7 *r* > 0.65 between cardiorespiratory parameters derived at hospital and at home was also found.

**Conclusion:** Autonomic function, as measured by HRV, differs according to MS phenotype. Autonomic response to walk tests may be useful for assessing clinical outcomes, mainly in the progressive stage of MS. Participants with larger changes in HRM are able to walk longer distance, while reduced ventilatory function during and after walk test performance is associated with higher fatigue and disability severity scores. Monitoring of disorder severity could also be feasible using ECG-derived cardiac and respiratory parameters recorded with a wearable device at home.

## 1 Introduction

Multiple Sclerosis (MS) is a chronic inflammatory disorder characterized by demyelination of the nerve fibers in the human Central Nervous System (CNS) (Faissner et al., 2019). Progressive neurodegeneration owing to chronic demyelination causes varied neurological deficits, which may considerably reduce the quality of life in people with MS (pwMS) (McGinley et al., 2021). The high prevalence of MS (2.8 million people in 2020) together with the relatively young onset age (between 20 and 40 years) have a significant impact on personal and societal costs (Wallin et al., 2019; Walton et al., 2020).

Although the exact cause of MS remains uncertain, it is considered an inflammatory autoimmune disorder that is influenced by both genetic and environmental risk factors (Baecher-Allan et al., 2018). A general consensus is that the disorder begins with inflammatory lesions (known as focal plaques) due to the dysregulation of the Blood-Brain Barrier (BBB) (Mahad et al., 2015). The disruption of the BBB increases the trans-endothelial migration of activated leukocytes into the CNS, which can initiate inflammatory responses by releasing pro-inflammatory cytokines (Ortiz et al., 2014). Furthermore, the sympathetic and parasympathetic branches of the Autonomic Nervous System (ANS) play a key role in the bidirectional communication between the immune system and the CNS (Racosta and Kimpinski, 2016). Sympathetic influence on immune response can be direct, *via* adrenoceptors on immune cells, or indirect, through innervation of major immune organs (Pongratz and Straub, 2014). The parasympathetic division of the ANS, through the activation of the efferent vagus nerve, inhibits pro-inflammatory cytokine release and protects against systemic inflammation (Pavlov and Tracey, 2012). Impairment of the ANS can either trigger inflammatory states or may fail to activate anti-inflammatory mechanisms, causing a vicious neuroinflammatory cycle that amplifies tissue damage (Findling et al., 2020).

Dysfunction of the ANS is being increasingly recognized as an important aspect of MS, with an estimated prevalence of 45%–84% (Racosta and Kimpinski, 2016). The main clinical manifestations of autonomic disturbances are associated with abnormal cardiovascular control (Findling et al., 2020), aberrant thermoregulation (Christogianni et al., 2018), gastrointestinal symptoms (Aghaz et al., 2018), and pelvic organ dysfunction (Aharony et al., 2017). Retrospective studies have shown that autonomic symptoms may be a key player in both prodromal phase and development of clinically established MS (Disanto et al., 2018; Almeida et al., 2019; Skorić et al., 2019). Autonomic dysregulation is also implicated in other common MS-related symptoms, such as fatigue (Ayache and Chalah, 2017; Capone et al., 2020), sleep disturbances (Hensen et al., 2018; Sakkas et al., 2019), and clinical depression (Gold et al., 2005; Morris et al., 2018). Therefore, these findings highlight the importance of monitoring the ANS dysfunction in people with MS.

The functionality of the ANS is often assessed based on changes in cardiovascular parameters during diagnostic tests including, Valsalva maneuver, deep breathing, and tilt-table test (Ziemssen and Siepmann, 2019). The variation in ANS activity, which is known as autonomic reactivity, is particularly important since it reflects the ability of an individual to cope with a challenging situation (Carnevali et al., 2018; Kontaxis et al., 2020). In order homeostasis to be maintained, cardiovascular parameters, such as Heart Rate (HR), are constantly changing. Short-term and long-term variations of HR, the so-called Heart Rate Variability (HRV), have emerged as the most valuable non-invasive measure of ANS function (Billman, 2011). Reduced contribution of the ANS to cardiac regulation, reflected as lower HRV, signifies pathological changes associated with autonomic dysfunction (Ernst, 2017). Reduced HRV in pwMS, compared to healthy subjects, has been reported in numerous studies (Racosta and Kimpinski, 2016; Findling et al., 2020). Moreover, the severity of HRV deficit appears to be associated with the progression of the disorder (Studer et al., 2017; Adamec et al., 2018). It has been hypothesized that autonomic imbalance towards a sympathetic dominance may have a pathogenic role in the development of MS, while parasympathetic dysfunction correlates with the progression of the disorder (Pintér et al., 2015; Racosta and Kimpinski, 2016).

However, there is no consensus regarding the presence of relation between HRV and the most common disorder characteristics, such as disability and fatigue. The most widely used disability rating scale in MS is the Expanded Disability Status Scale (EDSS) (Inojosa et al., 2020b), while the Fatigue Severity Scale (FSS) is one of the most well-known questionnaires evaluating the fatigue severity in pwMS (Krupp et al., 1989). The EDSS is mainly based on the evaluation of functional systems including ambulation, i.e., the maximal walking distance without rest, and several functional systems such as visual, sensory, and cerebral, among others (Kurtzke, 1983). The FSS evaluates both physical and cognitive aspects of fatigue and their interference in daily living (Beckerman et al., 2020). Previous studies have shown a significant positive relation between EDSS scores and HRV parameters reflecting sympathetic dominance (Studer et al., 2017; Zawadka-Kunikowska et al., 2020), while in (Damla et al., 2018), no significant association between HRV and EDSS was found. Although HRV tended to be different in pwMS with and without fatigue, no significant correlation with FSS scores has been observed (Flachenecker et al., 2003; Sander et al., 2019). The absence of consensus regarding the relation of HRV with these MS-related symptoms could be partially explained by the subjective nature of the questionnaires (van Munster and Uitdehaag, 2017). For instance, the EDSS assessment is subjective to the examining neurologist (Inojosa et al., 2020a), while perception of fatigue in pwMS can be increased by comorbid conditions such as sleeping disturbances and depression (Sternberg, 2017).

Among the most common MS-related characteristics are also walking limitations that can be used to complement other less objective clinical measures (Bethoux and Bennett, 2011; Kieseier and Pozzilli, 2012). The 6-Minute Walk Test (6 MWT), which records the maximum distance a patient walks in 6 min, has been the most commonly applied measure of walking capacity in MS (Cederberg et al., 2019). In routine clinical assessment, the 2-Minute Walk Test (2 MWT) can be considered a practical replacement for the 6 MWT, which often tends to be more burdensome for individuals with MS (Andersen et al., 2016; Scalzitti et al., 2018). Current research on the walking assessments is focused only on changes in the HR profile rather than fluctuations in HRV parameters. Emerging evidence suggests that, in pwMS, a lower increase in the HR induced by walking is associated with higher levels of disability (Bosnak-Guclu et al., 2012; Dalgas et al., 2014). Although blunted autonomic reactivity to endurance exercise or tilt-up table test has been previously reported in pwMS (Studer et al., 2017; Zawadka-Kunikowska et al., 2020; Gerasimova-Meigal et al., 2021), no relationship between reactivity indices and disability or fatigue scores was found (Gervasoni et al., 2018; Rampichini et al., 2020).

A possible explanation for the lack of consensus regarding the potential use of HRV may also be the heterogeneity of the applied methods and the influence of respiration in the estimation of HRV indices. Changes in respiratory parameters, such as respiration rate and tidal volume, can confound the relationship between ANS function and HRV indices (Grossman and Taylor, 2007). To the best of the authors’ knowledge, none of the previous studies has taken into account the effect of respiration on HRV in pwMS. Recent studies suggest that besides monitoring respiration when analyzing HRV indices, there is an increasing awareness of the need to include respiration parameters in MS (Rzepiński et al., 2022). Previous studies in pwMS have shown that autonomic imbalance could influence breathing control and upper airway muscle activity (Hensen et al., 2018). In (Heine et al., 2016), a significant association between disorder severity and cardiopulmonary fitness was found. Respiratory dysfunction frequently occurs in people with advanced MS contributing significantly to morbidity and mortality (Tzelepis and McCool, 2015).

In order to monitor deterioration in pwMS, a continuous monitoring of clinical state is required. Remote measurement technologies (RMT), including a variety of sensors, can actively and passively collect numerous data that can assess symptom severity and progression, or response to treatment (White et al., 2022). With the advances in RMT over the last few decades, it could be now possible to provide real-time objective multidimensional indications of patients’ clinical symptoms (Malasinghe et al., 2019). A growing number of studies have explored the feasibility and potential effectiveness of RMTs in measuring a variety of physiological parameters among pwMS (Bradshaw et al., 2017; Alexander et al., 2020). The vast majority of RMTs are employed for non-invasive measurement of physical activity, while wearable sensors measuring cardiac activity still require further research before being included as tools for assessing autonomic dysfunction among pwMS (Fuller et al., 2020; Xiang and Bernard, 2021).

In this study, changes in HRV and respiratory parameters derived from the ECG induced by walk tests are analyzed for assessing clinical outcomes in pwMS. Correlation analyses with clinical markers of disorder severity and progression are performed. The main hypothesis tested here is that stress response to walk tests quantified by autonomic reactivity indices and respiratory parameters are useful for assessing clinical outcomes. This study also explores the feasibility and acceptability of home-based monitoring in pwMS.

## 2 Materials and methods

### 2.1 Dataset

The data used in this study were collected as part of the Remote Assessment of Disease and Relapse-Central Nervous System (RADAR-CNS) research programme^1^. RADAR-CNS is an IMI2 project that aims to develop new ways of measuring disorders related to CNS, such as MS, using remote monitoring technologies, i.e., wearable devices and mobile sensors. The MS work package of RADAR-CNS, which consists of multiple clinical sites spread across several European countries, started recruiting participants in June 2018. Since then, active (questionnaires and physical tests), and passive monitoring strategies (wearable and smartwatch devices) have been in place by three sites: Ospedale San Raffaele in Milan, Italy, Vall d’Hebron Institut de Recerca in Barcelona, Spain, and the University Hospital Copenhagen, Rigshospitalet in Copenhagen, Denmark (Ranjan et al., 2019). Outcome measures in pwMS were assessed every 3 months during clinical visits in the hospital, while the phenotype of the disorder, i.e., either relapsing-remitting MS (RRMS) or secondary progressive MS (SPMS), was assessed at the first clinical visit. In each clinical visit, participants underwent an experimental protocol that consists of three stages: (a) a Basal stage (B) where the subjects were instructed to relax without speaking during 5 min, (b) a Test stage (T) during which, the individuals were performing the 2 MW T, and (c) a Recovery stage (R) where the subjects were supposed to achieve a resting state remaining silent during 5 min. During the whole protocol, an ECG signal was continuously recorded at a sampling frequency of 125 Hz for each participant using the wearable device Bittium Faros 180^2^. Note that other physical tests (6 MW T), and the filling of questionnaires either by the clinicians (EDSS) or the pwMS (FSS), were conducted before attaching the wearable device.

As of 1 November 2021, 206 pwMS had been enrolled in the RADAR-MS study, and a total of 384 clinical visits had been conducted (99, 58, 34, 9, 5, and 1 patient(s) had one to six clinical visits, respectively). Although ECG data during the 2 MW T (T) were available in all participants, 5min of ECG data during B and R were available in about 70% for each one of them. From all participants, 90 performed again a 2MWT wearing Faros at home the day after the clinical visit. Table 1 shows the demographic data and baseline clinical data for all participants. Approval of the protocol was obtained from hospitals’ ethical committees and informed consent obtained from all participants. This study was co-developed with service users in our Patient Advisory Board, who were involved in the choice of measures, the timing and issues of engagement. The inclusion criteria for this study were an age 18 or over, diagnosis of MS according to 2010 revisions to the McDonald criteria, RRMS or SPMS phenotypes, EDSS from 2 to 6, the ability to give informed consent for participation, the willingness and ability to complete self-reported assessments *via* smartphone, existing ownership of android smartphone, while exclusion criteria were the presence of any condition (physical, mental, or social) that is likely to affect the subject’s ability to comply with the protocol, and female subjects who are currently pregnant. For further information on the MS study and assessment schemes the interested reader is referred to (Dalla Costa et al., 2020).

**TABLE 1:** Mean ± standard deviation of demographic data and baseline clinical data.

	RRMS	SPMS
Number of subjects *n*	148	58
Sex (female/male)	94/54	33/25
Age, years	45.5 *±* 9.8	49.4 *±* 9.3
BMI, kg/m^2^	24.3 ± 4.9	25.3 ± 4.6
MS duration, years	14.9 *±* 7.9	19.8 *±* 10.5
MS progression, years		4.8 *±* 3.8
FSS (*n*)	4.4 ± 1.7 (135)	4.8 ± 1.3 (55)
EDSS (*n*)	2.9 *±* 1.1 (140)	4.0 *±* 1.2 (56)
2MWT, m (*n*)	123.8 *±* 22.8 (101)	100.5 *±* 26.1 (29)
6MWT, m (*n*)	402.1 *±* 66.6 (143)	350.3 *±* 90.7 (57)
MS medication *n* (%)		
Fingolimod	24 (16%)	9 (15%)
Natalizumab	12 (8%)	10 (17%)
Teriflunomide	20 (13%)	7 (12%)
Interferon-beta	22 (15%)	5 (8%)
Glatiramer acetate	17 (11%)	5 (8%)
Other	38 (25%)	14 (24%)
Co-morbidities *n* (%)		
Depression	6 (4%)	6 (10%)
Blood pressure disorders	5 (3%)	4 (7%)
Thyroid disorder	5 (3%)	3 (5%)
Heart diseases	2 (1%)	2 (3%)

Statistically significant differences are indicated in bold text.

MS, multiple sclerosis; RRMS, Relapsing–Remitting Multiple Sclerosis; SPMS, secondary progressive multiple sclerosis; BMI, body mass index; FSS, fatigue severity scale; EDSS, expanded disability status scale; 2MWT, 2-Minute Walk Test; 6MWT, 6-Minute Walk Test.

### 2.2 Heart rate variability and respiration indices

In this section the estimation of ECG-derived cardiorespiratory parameters that reflect ANS function is described. However, surface measurements of heart activity may include various types of noise, and thus ECG should be first preprocessed. To remove the baseline wander, the ECG signal was filtered using a forward-backward filter with cut-off frequency of 0.5 Hz. To reduce the effect of jitter into HRV parameters, the ECG was up-sampled from 125 to 1,000 Hz (Malik et al., 1996). Then, a wavelet-based detector was applied to identify the position of the QRS-complexes (Martínez et al., 2004). HRV was generated from the beat occurrence time series based on the integral pulse frequency modulation (IPFM) model, which accounts for misdetections and the presence of ectopic beats (Bailón et al., 2010). The IPFM model assumes that ANS modulation on the sinoatrial node can be represented by a band-limited signal. To derive spectral parameters, the modulating signal was sampled at 4 Hz, and the power spectral density was obtained by applying Welch’s periodogram in segments of length 5 min. A Hamming window of 100 s (50% overlap), which offers high temporal resolution and it allows the robust estimation of the lowest frequency of interest (4 periods for 0.04 Hz), was selected.

From short-term recordings, two main spectral components are distinguished in the low-frequency (LF) band ([0.04, 0.15] Hz) and in the high-frequency (HF) band ([0.15, 0.4] Hz). HF spectral power is considered a measure of parasympathetic nervous system activity, mainly due to the respiratory sinus arrhythmia, while LF fluctuations in HRV have been suggested to represent both sympathetic and parasympathetic modulations (Malik et al., 1996). Note that in order HF spectral power to reflect parasympathetic activity, respiratory rate must lay in the HF band. However, sometimes during exercise respiratory rate may be higher than the upper limit of the HF band. Hence, the extended HF band (eHF), which goes up to half the mean HR (*f*_HR_), i.e., the Nyquist frequency for HRV signals, is preferred (Bailón et al., 2007). Thus, the normalized LF power, i.e., the relative value of the LF power in proportion to the total (LF and eHF) power, is calculated for estimating sympathovagal balance (SB). Note that for the correct estimation of SB, respiratory rate must not lay in the LF band. In this study, since no respiration signal was recorded, respiratory parameters are estimated from the ECG. The ECG-derived respiration (EDR) signal, named slope range (Kontaxis et al., 2019), is used to obtain the mean respiratory rate (*f*_*r*_) from EDR spectra, and the standard deviation of the EDR signal (*σ*_*r*_), is used as surrogate of tidal volume (Varon et al., 2020). It should be noted that a median absolute deviation-based rule was implemented to remove outliers from the EDR signal before parameter estimation (Bailón et al., 2006).

**FIGURE 1:**
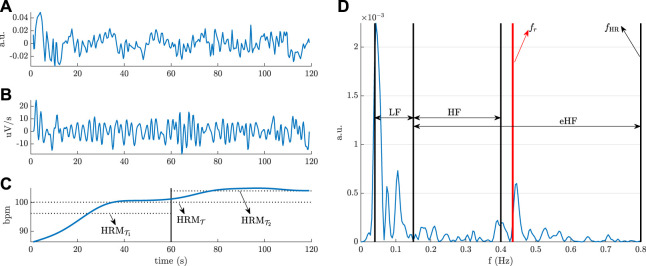
HRV analysis during the 2MWT **(A)** HRV signal, **(B)** EDR signal, **(C)** HR profile, and **(D)** HRV spectrum.

Besides HRM (HRM_*S*_), sympathovagal balance (SB_*S*_), and respiratory parameters ( $f_{r}^{S}$ , $\sigma_{r}^{S}$ ) that are calculated for each stage *S* (S=[B,T,R]) , HRM is also estimated during the first $\left({HRM}_{T_{1}}\right)$ and second minute $\left({HRM}_{T_{2}}\right)$ of the 2MWT in order to study the HR profile. The intra-subject difference between HRM parameters during test ( ${HRM}_{T}$ , ${HRM}_{T_{1}}$ , ${HRM}_{T_{2}}$ ) and either basal $\left({HRM}_{B}\right)$ or recovery stage $\left({HRM}_{R}\right)$ are considered autonomic reactivity indices. The reactivity indices, denoted $\Delta {\left(HRM\right)}_{S_{1}}^{S_{2}}$ , are calculated by subtracting the value of the HRM in $S_{1}=\left[B,R\right]$ from that in $S_{2}=\left[T,T_{1},T_{2}\right]$ . Figure 1 illustrates an example of HRV analysis during the 2MWT.

### 2.3 Statistical analysis

Since a small percentage of subjects has measurements for more than two clinical visits, the statistical analysis is performed by averaging first measurements across multiple visits for each subject. Statistical analysis is conducted to assess differences in ANS regulation between groups of pwMS with different clinical outcomes. First of all, ANS function in pwMS with different phenotype (RRMS or SPMS) is considered. Participants with low (FSS <4) or high (FSS ≥4) fatigue scores (FSS_*L*_ or FSS_*H*_, respectively), and with low (EDSS ≤2.5) or high (EDSS ≥4.5) disability scores (EDSS_*L*_ or EDSS_*H*_, respectively), consist in groups of interest (Krupp et al., 1989; Manouchehrinia et al., 2017). Since there is no cut-off value for grouping pwMS based on 6MWT or 2MWT scores, the lower and upper quartiles are used. Participants are grouped into low or high 6MWT (6MWT_*L*_ or 6MWT_*H*_). For separating pwMS based on statistical distribution, the 6MWT is selected as it has a higher completeness (97%) compared to 2MWT (63%), see Table 1. Statistically significant group differences (RRMS vs. SPMS, FSS_*L*_ vs. FSS_*H*_, EDSS_*L*_ vs. EDSS_*H*_, and 6MWT_*L*_ vs. 6MWT_*H*_) are assessed with the two-sample *t*-test for independent samples, and the Mann-Whitney U non-parametric test when appropriate. Shapiro-Wilk and the two-sample F-test are conducted for ensuring normality and equality of variances, respectively. Differences in baseline demographic data are also evaluated.

Regression models based on a clinical measure (Phenotype, FSS, EDSS, 6MWT, 2MWT) with demographic variables (Sex, Age, BMI) as covariates are also used in order to determine significant predictors for autonomic variables. Partial correlation (Pearson *r*) analyses controlling for demographic variables (Sex, Age, BMI) are carried out for testing bivariate associations between the most significant ANS parameters and clinical outcomes. Since ANS function might differ according to the phenotype of the disorder, correlation analyses are also conducted separately for the populations of RRMS and SPMS. Finally, correlation coefficients between ANS parameters derived at hospital and at home are calculated to test the feasibility of RMT to monitor disorder severity in pwMS outside clinical settings. The significance threshold in this study is set to *p* < 0.05.

## 3 Results

**FIGURE 2:**
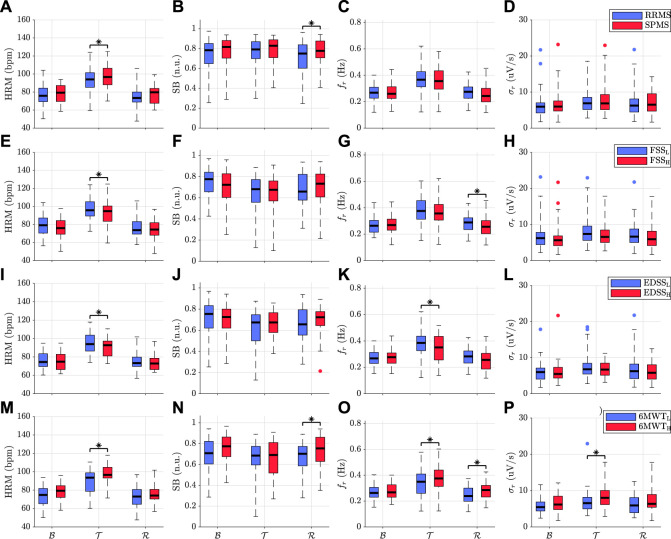
Boxplots of HRV and respiratory parameters for different groups of pwMS. Groups based on **(A–D)** phenotype, **(E–H)** fatigue severity, **(I–L)** disability level, and **(M–P)** walking capacity. Statistically significant group differences are denoted with an asterisk.

Figure 2 illustrates the boxplots of HRV and respiratory parameters for different groups of pwMS. Groups based on phenotype consist of 148 and 58 subjects for RRMS and SPMS group, respectively, while the groups of (low, high) scores for FSS, EDSS, and 6MWT, consist of (64, 131), (70, 35), and (51, 51) subjects, respectively. Regarding phenotype, SPMS group, compared to RRMS, shows statistically significant higher HRM during walk test (Figure 2A) and increased sympathovagal balance after test execution (Figure 2B). Participants with low fatigue scores reached higher HRM level during T and respiratory rate during R than those with higher fatigue scores (Figures 2E, G). In relation to disability, EDSS_*L*_ group, compared to EDSS_*H*_, exhibits also higher HRM during test execution (Figure 2I), as well as higher $f_{r}^{T}$ (Figure 2K) $\sigma_{r}^{R}$ (Figure 2L), suggesting that exercise may induce significant changes in respiratory system. Finally, pwMS who were able to walk longer distance (6MWT_*H*_) show higher ${HRM}_{T}$ and $\sigma_{r}^{T}$ (Figure 2M,P), that implies a greater cardiopulmonary effort compared to pwMS in 6MWT_*L*_ group. The same holds for the respiratory rate that remained higher in 6MWT_*H*_ group even after task execution (Figure 2O). Sympathetic dominance was statistically significant higher in 6MWT_*H*_ group during recovery period (Figure 2N). Furthermore, reactivity indices (not shown) were found to be statistically different between the groups of 6MWT, with higher reactivity to be associated with pwMS who were able to walk longer distance. It should be also noted that only a small percentage of about 3% of SB at each stage was excluded from the analysis because respiratory rate was laying in the LF band.

Significant predictors of ANS parameters in pwMS are presented in Table 2. Regression analysis results confirm that MS phenotype is an independent predictor for ${SB}_{R}$ besides the significant effect of age on sympathovagal balance. The FSS score, and the sex variable, were identified as independent predictors for $\sigma_{r}^{T}$ , while 2MWT, and 6MWT scores were statistically significant predictors for HRM reactivity measured with $\Delta {\left(HRM\right)}_{R}^{T_{2}}$ .

**TABLE 2:** Regression models to determine ANS parameters by clinical features.

Dependent variables	Independent variables	BETA (*β*)	SE	*t*	*p*-value
${SB}_{R}$	Phenotype	0.05	0.02	2.34	< 0.05
*R* = 0.75; *R* ^2^ = 0.56	Age	−0.003	0.001	−2.64	< 0.01
*F*(4) = 3.66; *p* < 0.01					
$\sigma_{r}^{T}$	FSS	−0.49	0.14	−3.44	< 0.001
*R* = 0.75; *R* ^2^ = 0.56	Sex	−1.55	0.48	−3.44	< 0.01
*F*(4) = 8.59; *p* < 0.001					
$\Delta {\left(HRM\right)}_{R}^{T_{2}}$	2MWT	0.08	0.03	2.66	< 0.01
*R* = 0.77; *R* ^2^ = 0.59					
*F*(4) = 3.63; *p* < 0.01					
$\Delta {\left(HRM\right)}_{R}^{T_{2}}$	6MWT	0.04	0.01	4.52	< 0.001
*R* = 0.80; *R* ^2^ = 0.64					
*F*(4) = 7.48; *p* < 0.001					

FSS, fatigue severity scale; 2MWT, 2-Minute Walk Test; 6MWT, 6-Minute Walk Test; ${SB}_{R}$ , sympathovagal balance at recovery phase; $\sigma_{r}^{T}$ , standard deviation of EDR signal during walk test; $\Delta {\left(HRM\right)}_{R}^{T_{2}}$ , change in HRM from second minute of 2MWT to recovery phase; *R*, the correlation between the predictor and the response variable; *R* ^2^, coefficient of determination; *F*, F-test statistic, BETA (*β*), the slope of regression line; SE, standard error between the points and the regression line; *t*, *t*-test statistic.

**FIGURE 3:**
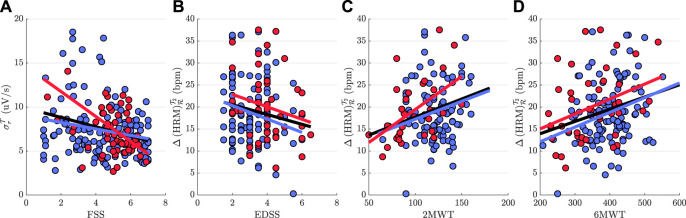
Scatter plots of the most significant ANS parameters for each clinical outcome measure **(A)** FSS, **(B)** EDSS, **(C)** 2MWT, and **(D)** 6MWT. A blue and red line are fitted to the data of RRMS and SPMS group, respectively, while a black line is fitted to the data without taking into account the phenotype of the disorder.

Results of partial correlation analyses are summarized in Table 3. It can be seen that, analyzing all pwMS without taking into account the phenotype of the disorder, there is a weak association between ANS parameters and outcome measures. In particular, a moderate positive correlation between 6MWT and reactivity indices of HRM, implies that higher autonomic reactivity is associated with pwMS who were able to walk longer distance. The increase of HRM during the last minute of the 2MWT with respect to recovery stage $\left(\Delta {\left(HRM\right)}_{R}^{T_{2}}\right)$ shows the best results (*r* = 0.36) (*r* = 0.32). Regarding EDSS, no significant association was found, while less-fatigued pwMS show a larger cardiopulmonary effort during the walk test ( ${HRM}_{T}$ , $\sigma_{r}^{T}$ ). However, differences in ANS regulation between people with RRMS and SPMS may blur the association of ANS parameters with the clinical markers. Taking into account the phenotype of the disorder, results show a strong positive correlation in people with SPMS between reactivity indices of HRM $\left(\Delta {\left(HRM\right)}_{R}^{T_{2}}\right)$ and 6MWT or 2MWT scores, yielding *r* = 0.50 and *r* = 0.51 (*r* = 0.51and *r* = 0.60), respectively. Moreover, a higher respiratory rate after test execution $\left(f_{r}^{R}\right)$ was associated with better walking capacity, yielding *r* = 0.40 (2MWT) and *r* = 0.29 (6MWT) (*r* = 0.48 (2MWT) and *r* = 0.33 (6MWT). A negative correlation between $\sigma_{r}^{T}$ and FSS (*r* = −0.45) (*r* = 0.50) suggests a higher tidal volume during walking in less-fatigued people with SPMS. Only for SPMS group there is significant correlation between autonomic reactivity and EDSS scores (*r* = −0.31) (*r* = 0.35). On the contrary, a weak positive relationship between sympathovagal balance $\left({SB}_{R}\right)$ and walk test scores was only found in RRMS group. Figure 3 illustrates the scatter plots of the most significant ANS parameters for each outcome measure.

**TABLE 3:** Partial correlation coefficient *r* between ANS parameters and clinical outcomes.

	MS				RRMS				SPMS			
	FSS	EDSS	2MWT	6MWT	FSS	EDSS	2MWT	6MWT	FSS	EDSS	2MWT	6MWT
${HRM}_{T}$	−0.22	n.s	n.s	0.24	−0.21	n.s	n.s	0.31	−0.34	−0.20	n.s	0.33
${SB}_{R}$	n.s	n.s	n.s	n.s	n.s	n.s	0.29	0.21	n.s	n.s	n.s	n.s
$\sigma_{r}^{T}$	−0.25	n.s	n.s	n.s	−0.22	n.s	n.s	n.s	−0.45	n.s	n.s	n.s
$f_{r}^{R}$	n.s	n.s	n.s	n.s	n.s	n.s	n.s	n.s	n.s	n.s	0.40	0.29
$\Delta {\left(HRM\right)}_{R}^{T_{1}}$	n.s	n.s	0.24	0.32	n.s	n.s	n.s	0.35	n.s	n.s	0.50	0.38
$\Delta {\left(HRM\right)}_{R}^{T_{2}}$	n.s	n.s	n.s	0.36	n.s	n.s	n.s	0.36	n.s	−0.31	0.51	0.50

^a^n.s., not statistically significant correlation value.

MS, multiple sclerosis; RRMS, Relapsing–Remitting MS; SPMS, Secondary Progressive MS; FSS, fatigue severity scale; EDSS, expanded disability status scale; 2MWT, 2-Minute Walk Test; 6MWT, 6-Minute Walk Test; ${HRM}_{T}$ , heart rate mean during walk test; ${SB}_{R}$ , sympathovagal balance at recovery phase; $\sigma_{r}^{T}$ , standard deviation of EDR signal during walk test; $f_{r}^{R}$ , respiratory frequency at recovery phase; $\Delta {\left(HRM\right)}_{R}^{T_{1}}$ , change in HRM from first minute of 2MWT to recovery phase; $\Delta {\left(HRM\right)}_{R}^{T_{2}}$ , change in HRM from second minute of 2MWT to recovery phase.

**FIGURE 4:**
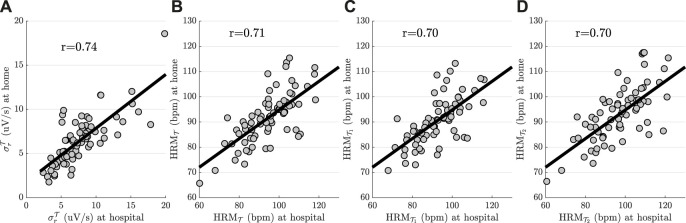
Scatter plots of the most significant ANS parameters derived during 2MWT performed at hospital and at home. **(A)** $\sigma_{r}^{T}$ , **(B)** ${HRM}_{T}$ , **(C)** ${HRM}_{T_{1}}$ , and **(D)** ${HRM}_{T_{2}}$ .

Finally, ANS parameters derived at home and at clinical visit while participants were performing the 2MWT, including $\sigma_{r}^{T}$ , ${HRM}_{T}$ , ${HRM}_{T_{1}}$ , and ${HRM}_{T_{2}}$ , showed a significant association, yielding *r* = 0.74, *r* = 0.71, *r* = 0.70, and *r* = 0.70 *r* = 0.69, *r* = 0.68, *r* = 0.67, and *r* = 0.70, respectively (Figure 4). This strong correlation implies that RMT could be used for monitoring disorder severity in pwMS, complementing thus, periodic evaluations performed at hospital.

## 4 Discussion

In this study the association between ANS response to walk tests and outcome measures in pwMS is investigated. Autonomic function is assessed with HRV and respiratory parameters derived from a wearable device, while MS-related symptoms, including fatigue severity, disability level, and walking capacity, are evaluated with rating scales and walk tests performed during periodical clinical visits. The feasibility of ANS parameters to provide a home-based monitoring of clinical markers is also explored.

HRV analysis can be used to assess the dynamic balance between the sympathetic and parasympathetic systems, allowing to infer the autonomic function. Results show that HRV parameters differ among participants depending on the MS phenotype. People with SPMS, compared to RRMS, show higher HRM while performing walk tests and larger SB after test performance (Figures 2A,B). Further statistical analyses revealed that the phenotype of MS was a significant predictor of sympathovagal balance (Table 2). Increased sympathetic dominance is associated with autonomic imbalance, which is often reported to be more pronounced in the progressive variant of the MS (Zawadka-Kunikowska et al., 2020; Gerasimova-Meigal et al., 2021). These results are in agreement with previous studies which found that pwMS exhibit higher HR levels during exercise compared to healthy subjects (Savci et al., 2005), and more prominent differences in autonomic tone during recovery phase (Gervasoni et al., 2018). Anticipatory stress dynamics prior to test execution could be implicated in the absence of significant differences between MS subgroups during basal conditions (Juster et al., 2012; Adamec et al., 2018; Reynders et al., 2020).

Autonomic dysregulation, besides the key role it plays in the progression of the disorder, it is also involved in the vast majority of MS-related symptoms (Findling et al., 2020). Results show that pwMS with low FSS and EDSS scores reach higher HRM values during walk tests (Figures 2E,I). Greater fatigue and disability severity in pwMS may be associated with loss in the capacity to increase cardiovascular output during exercise (Capone et al., 2020). Lower HR in moderate compared to mild disability group of pwMS at the end of walk tests was also observed in (Bosnak-Guclu et al., 2012). Similar to patients with chronic fatigue syndrome, HR was lower during exercise tests relative to control subjects (De Becker et al., 2000; Nelson et al., 2019). Furthermore, reduced cardiovascular output can lead to less ventilatory efficiency (Inbar et al., 2001; Javierre et al., 2010). This is in accordance with the lower respiratory rates ( $f_{r}^{R}$ , $f_{r}^{T}$ ) attained by pwMS with higher fatigue and disability severity scores (Figures 2G,K). Furthermore, a higher FSS score was a significant predictor of decreased standard deviation of the EDR signal measured with $\sigma_{r}^{T}$ (Table 2).

Abnormalities in cardiac and/or ventilatory function can limit exercise performance capacity in pwMS (Wens et al., 2016). Participants who were able to walk longer distance (6MWT_*H*_) exhibit higher ${HRM}_{T}$ , $f_{r}^{T}$ , and $\sigma_{r}^{T}$ values during walk tests (Figure 2M,O,P), implying a greater cardiopulmonary effort than pwMS in 6MWT_*L*_ group. An increased respiratory rate and sympathetic dominance in 6MWT_*H*_ group is also observed after test execution (Figure 2N,O). A significantly shorter distance walked by pwMS compared to healthy individuals was previously reported in (Savci et al., 2005), while cumulative evidence supports that changes in cardiorespiratory fitness outcomes during exercise are the most sensitive to detect worsening in pwMS (Paltamaa et al., 2008; Heine et al., 2016; Madsen et al., 2019). These data thus seem to indicate that the cardiorespiratory system is stressed when a walk test is performed by pwMS (Dalgas et al., 2014).

Autonomic response to stress can provide valuable information regarding the monitoring of clinical outcomes in MS (Briones-Buixassa et al., 2015; Rzepiński et al., 2022). Besides changes from baseline to stress exposure, the length and persistence of stress reactions following the demanding challenge should be considered (Juster et al., 2012). The findings of this study reveal that walked distance and HRM reactivity indices have a positive relationship (Table 3). The increment of HRM during the last minute of the 2MWT with respect to recovery stage shows the best performance (*r* = 0.36) (*r* = 0.32) in pwMS (RRMS and SPMS). Furthermore, 2MWT and 6MWT scores were found as significant predictors of increased HRM reactivity measured with $\Delta {\left(HRM\right)}_{R}^{T_{2}}$ (Table 2). These results imply that pwMS with larger autonomic reactivity were able to walk longer distance. Reduced autonomic reactivity in pwMS, compared to healthy subjects, has been previously documented (Gerasimova-Meigal et al., 2021). Moreover, blunted autonomic reactivity was found to be related with incomplete recovery from relapses (Studer et al., 2017). However, no relationship between reactivity indices and disability and/or fatigue severity scores is usually reported (Flachenecker et al., 2003; Gervasoni et al., 2018; Sander et al., 2019; Rampichini et al., 2020).

The weak associations between outcome measures and ANS parameters might be attributed to differences in autonomic regulation between people with RRMS and SPMS (Adamec et al., 2018). Correlation analyses show better results taking into account the phenotype of the disorder (Figure 3). Reactivity indices of HRM and walk test scores, in particular, show a strong positive association in people with SPMS, yielding (*r* = 0.50 and *r* = 0.51) (*r* = 0.51 and *r* = 0.6) for 6MWT and 2MWT scores, respectively (Table 3). In (Hansen et al., 2013), walking capacity in pwMS was also found to be highly correlated with the impairment in adapting HR. Moreover, results show that pwMS who were able to walk longer distance reach higher respiratory rate during recovery, while less-fatigued pwMS show a larger cardiopulmonary effort during walk test ( ${HRM}_{T}$ , $\sigma_{r}^{T}$ ). Previous studies reported that ventilatory dysfunction in pwMS was related to worse exercise tolerance (Hansen et al., 2015). Regarding disability, pwMS with lower EDSS values show higher reactivity indices of HRM, while other studies have reported a significant association with SB (Shirbani et al., 2018). In this study, only for RRMS group there is a significant correlation between SB and walk test scores.

Inconsistencies regarding the link between disorder severity and SB can be muddled by changes in respiration, since the beat-to-beat fluctuation in HR at the frequency of the respiratory cycle, which is known as the respiratory sinus arrhythmia component, is mediated by the parasympathetic branch of the ANS (Billman et al., 2015; Ziemssen and Siepmann, 2019). In this study, the influence of respiration on SB estimation is taken into account by tracking the respiratory rate and by modifying the classical HF band in HRV analysis. Beyond the heterogeneity of the applied methods, the assessment of ANS functionality using different autonomic tests could lead to discrepancies regarding the role of autonomic reactivity in MS. For instance, contradictory results have been reported about autonomic responses to mental and orthostatic stress (Studer et al., 2017; Vlcek et al., 2018; Imrich et al., 2021), while walk tests and deep breathing protocols may enhance differences in ANS regulation between pwMS and healthy subjects (Paltamaa et al., 2008; Wens et al., 2016; Gerasimova-Meigal et al., 2021).

Walk tests may be one of the most straightforward procedures for inducing autonomic changes, not only in clinical settings but also at home (Singh et al., 2014). With the development of RMTs, such as, smartphones, wearable sensors or home-based devices, ANS parameters can be monitored remotely Malasinghe et al. (2019). Strong correlation between ANS parameters derived at home and at clinical visits during the performance of walk tests (Figure 4) implies that RMTs can be used for home-based monitoring of disorder severity in pwMS. Aside from complementing clinical assessments, reducing clinic contact allows also pwMS who would not normally engage in studies to participate (Owens, 2020). Furthermore, the feasibility of RMT to detect small changes in impairment or functional improvement on a more regular basis could accurately represent long-term changes in the clinical state of pwMS (Alexander et al., 2020).

Although RMTs offer a lot of potential for research, they do have important limitations. Many technologies have a high initial engagement level that fades quickly if usage is not constantly monitored after the initial enthusiasm and commitment of users (Dorsey et al., 2017). In this study, only a small subset of subjects (about 60%) performed walk tests at home. Unintentional walk testing, which does not modify casual activity habits, may increase the number of pwMS who are less motivated to perform such tests for extended periods. According to a recent study, unintentional walk testing has been shown to be practical and useful for assessing walking capacity in free-living activities (Sokas et al., 2021). Another important limitation is the interpretation of SB during walk test. Despite vagal withdrawal, hyperpnea could entail a significant increase in HF power due to the mechanical effect of respiration on HRV (Cottin et al., 2008). Hence, a rise in sympathetic tone, and consequently in SB, might be blurred when respiratory rate and respiratory pattern are not taken into account. A better assessment of the SB could be achieved by removing respiratory influences from HRV (Varon et al., 2018).

Apart from monitoring respiration while assessing HRV indices, the need of evaluating respiratory function in pwMS is becoming increasingly apparent. Recent studies suggest that pwMS are at increased risk for sleep disturbances (Braley and Boudreau, 2016; Sakkas et al., 2019). Intermittent hypoxia produced by apneas during the night might enhance oxidative stress and aggravate MS neurodegeneration (Hensen et al., 2018). In addition to being a trigger for an acute MS exacerbation, sleep disorders contribute significantly to fatigue and other chronic MS symptoms (Braley and Boudreau, 2016; Sahraian et al., 2017). Thus, respiratory function should be assessed early in the illness course so that rehabilitation can be planned to prevent respiratory complications and improve quality of life in pwMS (Muhtaroglu et al., 2020). It should be also noted that autonomic tests assessing respiratory function (e.g., deep breathing test) (Rzepiński et al., 2022), could be more suitable instead of walk tests to quantify autonomic response in people with SPMS who often suffer from limited ambulation (Wens et al., 2016). Screening for both ventilatory and cardiac function could open a window of opportunities for the early diagnosis of the progressive stage of MS that is often established retrospectively and delayed (Inojosa et al., 2021). Finally, confirming findings of previous studies, both clinical outcomes and demographic characteristics (Table 2) may be also considered as significant predictors of autonomic function in pwMS Rzepiński et al. (2019). Thus, further research should be conducted for people with primary progressive MS, including a healthy control group as well, taking into account differences in more demographic predictors (e.g., fitness levels), and changes in medication or other disease modifying treatments (e.g., haematopoietic stem cell transplantation).

In conclusion, the findings of this study show that autonomic function as measured by HRV differs according to MS phenotype, with sympathetic dominance to be more pronounced in SPMS compared to RRMS. Autonomic response to walk tests quantified by autonomic reactivity indices and respiratory parameters are useful for assessing clinical outcomes mainly in the progressive stage of MS. Moreover, pwMS with larger autonomic reactivity, in particular, are able to walk longer distance, while reduced ventilatory function during and after walk test performance is associated with higher fatigue and disability severity scores. Finally, this study demonstrates that monitoring of disorder severity could be feasible using ECG-derived cardiac and respiratory parameters recorded with a wearable device at home.

## Data Availability

The datasets presented in this article are not readily available because of ethic requirements. Requests to access the datasets should be directed to the RADAR-CNS consortium and will be subject to local ethics clearances. Please email the corresponding author for details.

## Footnotes

1
https://www.radar-cns.org/

2
https://www.bittium.com/medical/bittium-faros
